# A bibliometric and systematic study on public policy, health, and culture in the transformation of gender equity

**DOI:** 10.3389/fsoc.2026.1800195

**Published:** 2026-05-01

**Authors:** Luis Guillermo Guamán-Llongo, Mónica Elizabeth Zea-Vera, Diana Carolina Macías-Vilela, Daniel Angulo

**Affiliations:** State University of Milagro, Milagro, Ecuador

**Keywords:** bibliometrics, gender equity, intersectionality, public policy, social justice, systematic review

## Abstract

**Introduction:**

Gender equity remains a central issue in contemporary research and public policy, shaped by structural, cultural, and institutional inequalities. Despite increased academic attention, the field continues to exhibit fragmentation across disciplines and persistent gaps, particularly in intersectional and Global South perspectives. This study aims to analyze the evolution, patterns, and thematic structure of scientific production on gender equity from an interdisciplinary perspective.

**Methods:**

A bibliometric review complemented with systematic elements was conducted following PRISMA 2020 guidelines. A total of 835 records were identified from Scopus and Web of Science, of which 129 peer-reviewed articles published between 1986 and 2025 met the inclusion criteria. Data were processed using R (version 4.4.2) for cleaning, filtering, and trend analysis, while VOSviewer (version 1.6.20) was used to map international collaboration networks and keyword co-occurrence patterns.

**Results:**

Findings reveal a sustained increase in scientific production, particularly after 2011, with peak publication levels in 2023. Citation patterns indicate concentrated impact in specific years, notably 2002, 2011, and 2018. The analysis highlights the predominance of Global North institutions, thematic concentration in public policy, health, labor, and education, and limited representation of emerging areas such as environmental justice and technological inclusion. Additionally, collaboration networks exhibit asymmetrical structures, with unequal participation across regions.

**Discussion:**

The study demonstrates that while research on gender equity has expanded quantitatively, it remains uneven in impact and scope. Persistent gaps in intersectionality, regional representation, and thematic diversification suggest the need for more inclusive and interdisciplinary approaches. These findings underscore the importance of strengthening collaborative networks, integrating diverse perspectives, and aligning academic production with transformative public policy strategies.

## Introduction

Gender equity is fundamental to achieving sustainable development, social justice, and the transformation of contemporary institutional structures (Franc et al., 2018; Daly, 2002; Seguino, 2011). Despite normative progress and the increasing integration of egalitarian discourses into global agendas, structural disparities persist, hindering the realization of substantive equality in the realms of labor, education, health, and politics (Benería, 2008; Yates, 1997).

An effective strategy for addressing this phenomenon is the implementation of public health initiatives with a gender perspective. Recent studies have demonstrated that social determinants of health affect men and women differently, thereby intensifying the disease burden in rural and marginalized communities (Franc et al., 2018; Cowling et al., 2014). These findings underscore the necessity for health systems that address structural inequalities.

Simultaneously, the recognition of care as a public good has instigated a paradigmatic shift in social policy frameworks. Scholars such as Daly (2002) and Benería (2008) have posited that the institutionalization of care is crucial for achieving comprehensive and equitable citizenship. More recent research has connected this domain to the precarious nature of female labor and the lack of effective work-life balance policies (Mun et al., 2024).

The cultural dimension significantly contributes to the perpetuation of inequalities. In contexts characterized by high levels of religiosity or stringent traditional norms, women encounter persistent obstacles to their participation in economic and political spheres (Seguino, 2011; Matthews, 2022). This symbolic influence is also apparent in education, labor, and politics, where it reinforces gender stereotypes and consolidates unequal roles.

Structural barriers, including occupational segregation, underrepresentation in leadership roles, and wage disparities, continue to persist even within public and academic sectors (Chakraborty, 2020; McGrath et al., 2022). In the realms of entrepreneurship and innovation, research has documented the undervaluation of female leadership, their limited presence in patent registries, and institutional biases that restrict access to financing (Welsh et al., 2017; Zetter et al., 2017; Méndez-Suárez et al., 2025).

Structural violence, encompassing both physical and symbolic dimensions, constitutes a significant impediment. In the context of Latin America, empirical studies have demonstrated that domestic violence adversely affects women’s mobility, employability, and autonomy (Contreras et al., 2023). Furthermore, the implementation of health policies with a gender focus encounters institutional resistance (Russi-Ardila, 2020).

Research on gender equity demonstrates a geographically uneven distribution in terms of scientific output. Countries in the Global North dominate influential academic networks, whereas perspectives from the Global South are underrepresented in high-impact forums (Forgues-Puccio and Lauw, 2021; Plomien, 2019). Furthermore, issues such as intersectional discrimination (Arzú, 2021) and the challenges faced by trans women (Naradech, 2023) necessitate approaches that go beyond traditional binary frameworks.

This study conceptualizes gender equity not as a single-domain issue but as a multidimensional phenomenon embedded within public policy frameworks and interacting with health systems, labor structures, and sociocultural norms. Specifically, this study focuses on how gender equity is addressed within public policy-related research domains, rather than treating all gender-related research indiscriminately. Therefore, the research adopts an interdisciplinary perspective to capture the complexity of gender equity beyond sector-specific analyses.

This study employs a critical, systematic, and interdisciplinary approach to examine recent scientific production on gender equity through a bibliometric analysis and systematic review. Its objective is to identify achievements, gaps, and challenges to inform the development of more inclusive and transformative public policies.

In this context, the present study sought to address the following research questions:

(1) What are the main trends and gaps in the scientific production of gender equity from an interdisciplinary perspective?
(2) How have public policy, health, and cultural approaches influenced the evolution and visibility of gender equity research?
(3) What patterns of international collaboration and thematic focus shape the current landscape of gender equity studies?

To answer these questions, this paper is structured as follows: Section 2 presents a Literature Review, and Section 3 describes the methodology, including the systematic search strategy and bibliometric techniques employed. Section 4 presents the main findings on productivity, impact, journal relevance, collaboration networks, and the thematic areas. Section 5 discusses the implications of these findings in broader academic and policy contexts. Finally, conclusions are presented in Section 6.

## Literature review

From a theoretical perspective, gender equity has been extensively conceptualized within feminist scholarship as a structural and multidimensional phenomenon. Intersectionality theory (Crenshaw, 1991) highlights how gender inequalities intersect with race, class, and other axes of oppression, challenging universalist approaches. Similarly, feminist policy analysis (Lombardo and Meier, 2008) emphasizes that public policies are not gender-neutral but embedded in power relations that shape their outcomes.

Walby (2020) further conceptualizes gender inequality as a systemic structure reproduced across economic, political, and cultural domains, while Benería (2008) and Daly (2002) underline the centrality of the care economy in sustaining gendered divisions of labor. These frameworks collectively provide the analytical foundation necessary to interpret gender equity beyond formal policy design, incorporating structural and symbolic dimensions.

Building on these theoretical foundations, gender equity has been extensively examined from various disciplinary perspectives, including public policy, health, and sociocultural studies. Seminal works such as Daly (2002) introduced the concept of care as a public good, positing that its institutionalization is crucial for achieving gender equality. Similarly, Benería (2008) connected the care crisis to international migration and labor precarity, highlighting the significance of economic justice. In the health sector, Franc et al. (2018) demonstrated how gender factors exacerbate disease burdens in rural communities, underscoring the importance of intersectional public health systems. From a cultural perspective, Seguino (2011) and Matthews (2022) illustrated that religious and traditional norms often constrain women’s public participation.

More recent studies have expanded these perspectives. McGrath et al. (2022) conducted a global survey of anthropological practices, revealing widespread labor precarization among women researchers. Méndez-Suárez et al. (2025) analyzed how perceptions of effort influence female entrepreneurship, uncovering structural and symbolic barriers. Lambert et al. (2021) examined the socio-economic impact of the COVID-19 pandemic on working mothers in France, reinforcing the urgency of gender-responsive public policies. Collectively, these studies underscore that gender equity remains a multidimensional challenge, shaped by persistent institutional, economic, and symbolic inequalities.

However, despite these advances, existing literature remains fragmented across disciplines, with limited integration between bibliometric evidence and theoretically grounded interpretations of gender equity.

This review establishes the foundation for the present study’s critical exploration of the evolution, impact, and geographical disparities in gender equity research.

## Methodology

This study employed a bibliometric design, a quantitative methodology extensively utilized to assess scientific output through indicators such as publication volume, citation count, and keyword frequency. Bibliometric analysis facilitates the mapping of research trends, intellectual structures, and collaborative networks across disciplines (Donthu et al. 2021). This provides a systematic and replicable framework for evaluating the evolution and impact of knowledge within a specific field.

Unlike previous studies that concentrated solely on bibliometric indicators within a single domain, such as education or public health, this study integrates bibliometrics with a systematic literature review in accordance with the PRISMA 2020 guidelines. This dual approach enables a more comprehensive analysis by combining quantitative metrics with qualitative insights, thereby capturing the breadth and depth of gender equity research. Furthermore, this study utilized advanced tools such as R (version 4.4.2) and VOSviewer to visualize international collaboration and thematic clusters, offering an interdisciplinary perspective that bridges policy, health, and sociocultural dynamics, which are often treated separately in prior analyses.

This study adopts a quantitative, non-experimental, descriptive, and retrospective approach, employing a bibliometric design to analyze scientific production on public policy, health, and culture in the context of gender equity transformation. Additionally, criteria from the Preferred Reporting Items for Systematic Reviews and Meta-Analyses (PRISMA 2020) protocol were incorporated to ensure transparency and rigor in the identification, selection, and evaluation of included documents (Page et al., 2021).

Although PRISMA 2020 guidelines were followed to ensure transparency in the identification and selection of studies, the present research is best understood as a bibliometric review complemented by systematic elements, rather than a full systematic review including formal quality appraisal.

### Information sources and search strategy

Scopus and Web of Science (WoS) were employed because of their comprehensive international coverage, high indexing quality, and robust analytical tools (Singh et al., 2025; Vasudevan et al., 2025). The Science Citation Index Expanded (SCI-EXPANDED) and Social Sciences Citation Index (SSCI) collections were used. Regional databases, such as SciELO or Google Scholar, were excluded because of their limitations in global thematic coverage and peer-review criteria.

For Scopus, the search equation applied was: TITLE-ABS-KEY((“public policy” OR “government policy”) AND (“gender discrimination” OR “gender inequality” OR “gender equity”)). For WoS, the search equation was TS = ((“public policy” OR “government policy”) AND (“gender discrimination” OR “gender inequality” OR “gender equity”)).

The search strategy was intentionally designed to capture the intersection between public policy, institutional frameworks, and gender equity, rather than the entire spectrum of gender studies literature. Therefore, the resulting dataset reflects a focused analytical domain. It is acknowledged that broader terms such as “gender mainstreaming,” “care policy,” or “gender-based violence” could expand the dataset, which represents an opportunity for future research.

Due to the interdisciplinary nature of gender equity, the search strategy allows the inclusion of studies related to health, labor, education, and social policy, as these domains represent key areas where public policy interventions materialize.

This approach ensures consistency between the conceptual framework and the empirical corpus analyzed, avoiding the exclusion of relevant policy-related evidence.

### Inclusion and exclusion criteria

Two types of criteria were applied: (1) Only original peer-reviewed journal articles were included, and (2) Records published in Spanish and English were selected. Duplicates and studies lacking complete information on geographical or thematic context necessary for the study’s objectives were excluded.

### Data extraction and cleaning procedure

**Figure 1:**
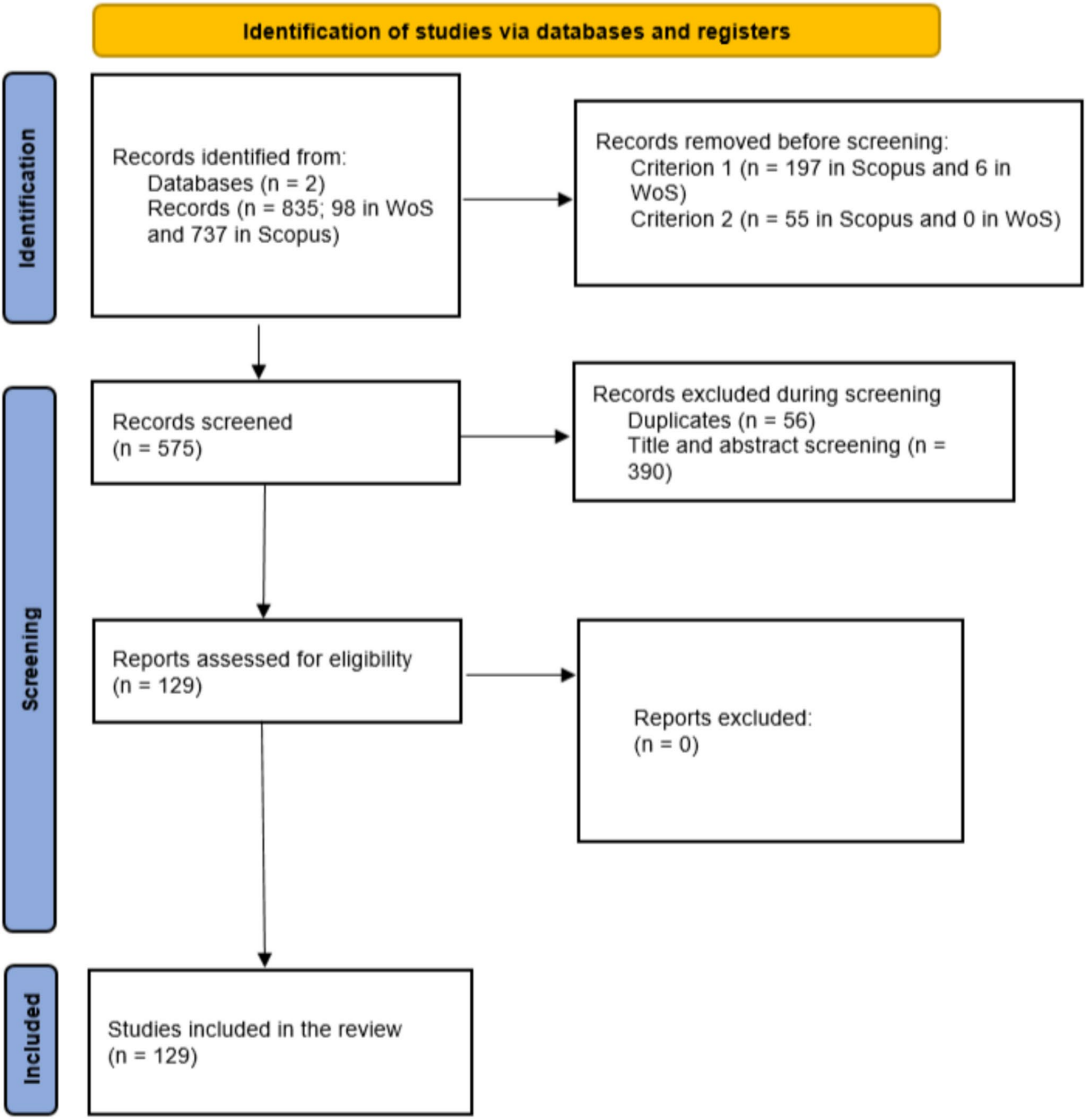
PRISMA flow diagram for study selection.

Data obtained from the search equations were exported in.csv format from Scopus and.xlsx format from Web of Science. A cleaning process was conducted to eliminate duplicates and irrelevant records. This involved reviewing titles and abstracts, and, in cases of uncertainty, consulting the full text of documents. To ensure transparency and systematization, the PRISMA flow diagram criteria were used, documenting the selection and exclusion phases in detail (Figure 1).

### Bibliometric analysis

The bibliometric analysis was conducted using R software (version 4.4.2). A combination of specialized packages was used for data processing and cleaning:

readxl: To import WoS records saved in.xls format.
data.table: To efficiently read and manage large Scopus files in.csv format.
dplyr: For data manipulation, including merging datasets, filtering records based on Boolean search terms, and selecting relevant variables.
openxlsx: To export cleaned and merged datasets in.xlsx format.
ggplot2 and gridExtra: To generate graphical representations of publication and citation trends over the analyzed period.

Bibliometric records from Web of Science and Scopus were integrated after standardizing titles to lowercase. Duplicates were identified and removed by comparing titles. A Boolean filter was applied to ensure thematic relevance, selecting only articles explicitly including key terms such as “Public Policy,” “Government Policy,” “Gender Equity,” and “Gender Equality” in the title or abstract.

Articles classified as reviews or exclusively bibliometric studies were excluded to maintain the empirical focus of the research. Productivity, measured as the number of publications, and impact, assessed through citations received, were synthesized by year and visualized using cumulative area charts and bar graphs, which highlighted emerging trends in the field.

Finally, VOSviewer (version 1.6.20) was used to generate co-occurrence maps of keywords and international collaboration networks. These visualizations provided a graphical representation of the structure and interconnections within the research field of gender equity.

## Results

A total of 835 records were identified (737 from Scopus and 98 from WoS). After removing duplicates and applying inclusion and exclusion criteria, 129 studies were selected for bibliometric analysis (Figure 1).

### Bibliometric analysis

#### Annual productivity and citations

The temporal analysis of scientific production on gender equity from 1986 to 2025 reveals progressive growth, with a notable increase starting in 2000. In the early decades, production was scarce and sporadic; however, since 2011, a sustained rise in annual publications is evident. This growth peaks in 2023, with 14 articles, marking a milestone in recent academic interest in this topic.

Citation analysis shows a more variable dynamic, with peaks of high influence in specific years. The year 2018 stands out as the most impactful, with 583 citations, followed by 2011 with 299 and 2002 with 205. This pattern suggests that certain works published in these years have significantly shaped the field’s development, possibly due to their focus on emerging issues or the introduction of innovative analytical frameworks widely referenced.

Notably, recent years like 2023 and 2024, despite high productivity with 14 and 6 publications, respectively, show low citation counts (20 and 1, respectively). This is consistent with the limited time elapsed since their publication, as academic citations tend to accumulate progressively.

Years with limited production but notable impact were also identified. For instance, in 1997 and 2002, only one article was published each year, accumulating 114 and 205 citations, respectively. These cases illustrate the potential of individual studies to generate significant impact, particularly when addressing conceptual gaps or proposing novel approaches.

**Figure 2:**
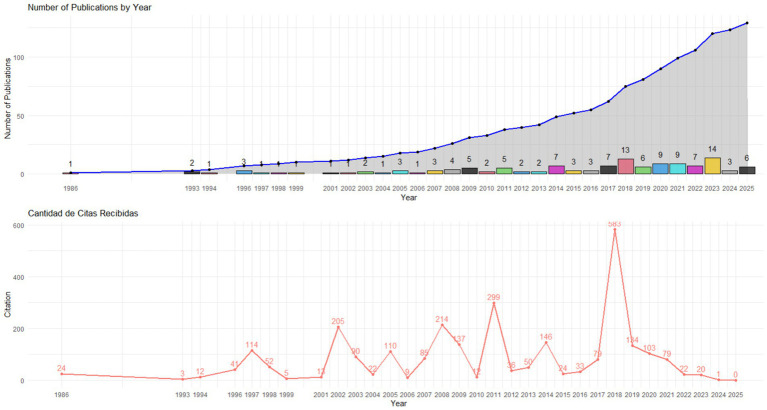
Annual evolution of publications and citations on gender equity.

In 2025, 6 publications were recorded with no reported citations, a result attributable to the year being recent or ongoing. Thus, the impact of these studies on academic literature remains to be evaluated (see Figure 2).

The analysis of the most cited articles within the bibliographic corpus reveals a wide diversity of approaches and contexts that enrich the multidimensional understanding of gender equity. These works, addressing themes such as public policy, the care crisis, religion, education, and health, provide foundational findings that contribute to designing more effective and context-specific strategies.

Among the most influential studies, Franc et al. (2018) stand out with over 300 citations. Though focused on brucellosis as a neglected zoonotic disease, it highlights how gender factors exacerbate the health burden in rural communities, underscoring the need for an intersectional approach in public health. In social policy, Daly (2002) introduces the concept of care as a public good, arguing that its institutionalization is essential for equal citizenship. This perspective has significantly impacted debates on redistributing reproductive work and providing public services.

From a sociocultural perspective, Seguino (2011) examines the influence of religion on gender equity, concluding that in highly religious contexts, patriarchal norms persist, hindering women’s participation in economic and political spheres. Benería (2008) analyzes the care crisis in Europe, linking it to migratory flows and female labor precarity, highlighting interconnections between migration policy, gender, and economic justice. In education, Yates (1997) problematizes the discourse on gender equity concerning boys, advocating for more inclusive and critical curricular frameworks.

Other relevant studies explore labor exclusion, structural violence, and political representation. Plomien (2019) evaluates the ambivalent effects of childcare policies in Europe, noting that their impact on gender equity depends on institutional design and implementation. Levasseur and Paterson (2016), through an analysis of apprenticeship programs in Canada, highlight inequalities in women’s access to technical jobs, even under inclusive policies.

Research focusing on the symbolic dimension of discrimination also offers significant insights. Phipps and Prieto (2021) historically reconstruct barriers faced by women leaders, noting that cultural imaginaries, beyond legal barriers, continue to undermine the legitimacy of female leadership. Similarly, Ariza-Ruiz et al. (2017) in Colombia demonstrate how menstruation-related stigmas negatively impact girls’ and adolescents’ rights to education and health, emphasizing the importance of integrating sexual and reproductive health into gender policies.

Collectively, these studies emphasize that gender equity cannot be addressed through a single thematic or institutional lens. The findings indicate that gaps persist not only due to the absence of regulations but also due to the reproduction of cultural, economic, and symbolic inequalities. Thus, the need for intersectional approaches that articulate public policies, social participation, structural reforms, and cultural transformations is highlighted to achieve significant and sustainable change.

### Scientific journals with the highest production

The analysis of journals with the highest number of publications reveals a notable concentration in a limited set of sources. Of the 32 journals identified in the 129 selected studies, Table 1 lists the nine most productive, identifying the main channels for scientific dissemination in gender equity from 2015 to 2025.

**Table 1:** Journals with the highest number of publications on gender equity.

‘Source title’	Cantidad	Citas
Contemporary Readings in Law and Social Justice	3	18
Cepal Review	3	7
Journal of Social Policy	2	212
Feminist Economics	2	189
International Entrepreneurship and Management Journal	2	31
Women in Management Review	2	29
Asian-Pacific Economic Literature	2	21
SAUDE E SOCIEDADE	4	15
Revista de Salud Publica	2	6

Among the journals with the highest volume of articles, Contemporary Readings in Law and Social Justice and Cepal Review lead with three publications each. However, their citation levels are relatively modest, with 18 and 7 citations, respectively, indicating moderate productivity but limited bibliometric impact. In contrast, other journals with an equal or lower number of publications show significantly higher impact. For example, the Journal of Social Policy, with only two articles, accumulates 212 citations, while *Feminist Economics*, also with two articles, records 189 citations. These data suggest that, despite lower publication volume, these journals exert considerable influence in the field.

Journals such as International Entrepreneurship and Management Journal and Women in Management Review contribute two publications each, accumulating 31 and 29 citations, respectively, reflecting intermediate relevance in productivity and impact. Meanwhile, journals like Asian-Pacific Economic Literature, SAUDE E SOCIEDADE, Saude e Sociedade, and Revista de Salud Pública show notable participation in volume but have a more limited impact, with fewer than 21 citations each.

The predominance of multidisciplinary journals or those specializing in social studies, economics, or public health underscores the cross-cutting nature of gender equity as a research subject. Additionally, the dominance of internationally scoped journals indicates that the most influential studies transcend local contexts and integrate into global debates on social justice, public policy, and sustainable development.

This analysis concludes that bibliometric impact is not necessarily correlated with publication volume. Certain journals, though less prolific, concentrate significant citations, establishing themselves as key nodes in scientific production on gender equity.

The analysis of the most relevant studies published in the 10 most productive scientific journals on gender equity highlights a wide diversity of thematic and methodological approaches, underscoring the multidimensional nature of the phenomenon. In Contemporary Readings in Law and Social Justice, works by Lăzăroiu et al. (2018) and others adopt a critical perspective to analyze power inequalities, highlighting how misogynistic violence and normative inequities continue to shape social and labor relations, even in contexts with legislative advances.

In Cepal Review, studies by Blofield and Martínez (2014) and Güezmes García et al. (2023) examine transformations in public care policies in Latin America, showing that despite institutional recognition of co-responsibility, women continue to disproportionately bear unpaid care responsibilities. This observation highlights the need for more inclusive and sustainable care models.

Research published in the Journal of Social Policy, such as the work of Javakhishvili and Jibladze (2018), suggests that the implementation of anti-gender violence policies in regions such as Georgia is often symbolic and hindered by a lack of commitment from responsible parties and entrenched patriarchal norms. Feminist Economics provides a critical examination of the economy’s role in perpetuating inequalities, with studies indicating that labor market structures disadvantage women in terms of wages and promotion opportunities (Benería, 2008; Gálvez-Muñoz et al., 2011).

The International Entrepreneurship and Management Journal documents low participation of women in innovative entrepreneurship, which is affected by gender stereotypes, institutional barriers, and limited access to capital (Welsh et al., 2017; Méndez-Suárez et al., 2025). Women in Management Review further explores this issue by analyzing the “glass ceiling” that restricts women’s access to executive positions in large corporations, particularly in developing countries (Shilton et al., 1996; Den Dulk et al., 1996). In the Asian-Pacific Economic Literature, studies by Cho et al. (2013) and Meng (1996) emphasize how social security policies in Asia have systematically excluded women from certain benefits, thereby perpetuating their economic vulnerability.

SAUDE E SOCIEDADE, focusing on public health, address the intersection of health and gender, highlighting the lack of access to tailored services and the invisibility of women’s specific needs in rural and marginalized urban areas. Finally, research in Revista de Salud Pública demonstrates how cultural practices, such as menstruation stigma, directly impact girls’ education in Latin America and how structural violence against women is reflected in health indicators.

Collectively, these studies support the findings of this study by illustrating that despite normative and discursive progress in gender equity, deep-seated resistance persists in social, cultural, and economic practices. The compiled evidence underscores the necessity for intersectional approaches that recognize diverse contexts and facilitate the development of more comprehensive, inclusive, and transformative public policies.

### International collaboration

The analysis of the international collaboration network in scientific production on gender equity reveals a markedly unequal distribution of country participation. A notable concentration of connections is observed in regions with greater academic infrastructure and access to research funding, particularly in North America, Western Europe, and certain emerging economies in Asia and Latin America. This distribution not only highlights the leadership of certain nations in the field but also the existence of consolidated networks among universities, research centers, and multilateral organizations collaborating jointly.

The most prominent nodes within the network are linked to countries with high publication volumes and substantial capacity to form strategic alliances, thereby serving as connectors between diverse regions. Recurrent patterns of bilateral collaboration have been identified, indicating a dynamic in which certain countries concentrate on editorial control, while others primarily participate as co-authors or secondary collaborators. This structure partially reflects asymmetries in the access to resources, data, and scientific publication platforms.

**Figure 3:**
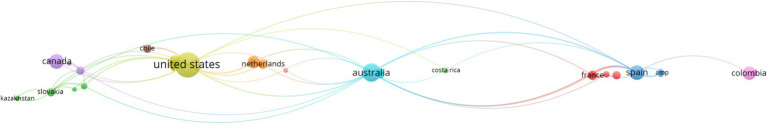
Map of international collaboration in gender equity research.

Despite these disparities, the existence of intercontinental collaborations signifies efforts to internationalize the discourse on gender equity, integrating diverse contexts that enhance comparative methodologies. Nevertheless, the underrepresentation of certain Global South countries within the network highlights the imperative to bolster local research capacities and advocate open science policies that facilitate more equitable participation in scientific production (see Figure 3).

The analysis of the reviewed studies highlights the relevance of international collaboration in scientific production on gender equity. The joint participation of institutions from North America, Europe, Asia, Africa, and Latin America has enriched both the thematic diversity and methodological depth of research, consolidating a multidimensional approach to the phenomenon.

Among the most impactful works, Franc et al. (2018), a collaboration between institutions in the United States, South Africa, Switzerland, and Mexico, analyzes how gender factors influence exposure and management of zoonotic diseases. Daly (2002), from Queen’s University Belfast, conceptualizes care as a public good, emphasizing its importance for equal citizenship. Seguino (2011), at the University of Vermont, examines the role of religion in perpetuating gender inequalities. Benería (2008), from Cornell University, studies the care crisis in relation to international migrations, providing evidence of female labor precarity.

Yates (1997), from Australia, problematizes gender equity in education, while Saharso (2003), in the Netherlands, analyzes the impact of cultural notions of tolerance on gender policies. Cowling et al. (2014), in India, delve into social determinants of health associated with gender inequalities. Cañibano et al. (2016), from Spain, investigate female labor mobility in academia, and Welsh et al. (2017), in the United States, explore perceptions of gender discrimination in entrepreneurship. Phipps and Prieto (2021), in the United Kingdom, address historical symbolic obstacles faced by women leaders.

Other relevant studies reinforce these perspectives. Chakraborty (2020), in India, documents persistent wage gaps between public and private sectors. Frías (2017), in Mexico, analyzes dynamics of intimate partner violence. Lambert et al. (2021), in France, evaluate the socioeconomic impacts of the pandemic on working mothers. McGrath et al. (2022) examine precarious working conditions of anthropologists globally. Matthews (2022), in Ghana, studies the political impact of women’s social movements. Arzú (2021), in Guatemala, addresses the intersection of racism and gender, while Naradech (2023), in Thailand, analyzes labor discrimination against trans women.

Forgues-Puccio and Lauw (2021) establish a relationship between higher gender equity and lower corruption levels internationally. Plomien (2019) evaluates the ambivalent effects of childcare policies in Europe. Zetter et al. (2017), in Mexico, investigate low female representation in patent registries. Linthon-Delgado and Méndez-Heras (2022), in Ecuador, decompose the causes of the wage gap. Hoyt and Kurtulus (2025), in the United States, analyze the impact of wrongful discharge laws on female employment. Méndez-Suárez et al. (2025), in Latin America, study how social perceptions of female effort limit entrepreneurship.

Other significant contributions include Mun et al. (2024), in the United States, examining the effect of parental leave on organizational inequality. Dekel and Elefant (2023), in Israel, analyze budget allocation in the arts and its gender impact. Contreras et al. (2023), in Chile, study the mobility of women affected by domestic violence, while in Colombia, Contreras et al. (2023) explore convergences between ecology and gender. Russi-Ardila (2020), also in Colombia, evaluates the legitimacy of equity policies in health institutions. Finally, Ariza-Ruiz et al. (2017), in Colombia, investigate the effects of menstrual stigma on rural girls’ education.

These studies demonstrate that international collaboration enhances analytical capacity, enables comparison of diverse realities, and strengthens theoretical and methodological frameworks on gender equity. They also highlight the need for intersectional approaches sensitive to local contexts and coordinated strategies to drive structural transformations beyond normative advances.

### Thematic area

The analysis of the thematic distribution of the examined studies reveals notable diversity in approaches to addressing gender equity. The predominant thematic areas focus on public policy, labor dynamics, health, and education, highlighting the multidimensional nature of this issue. These areas concentrate the majority of records, indicating they are the fields of greatest research interest and perceived need for institutional intervention.

At a secondary level, emerging themes such as gender-focused technological innovation, female leadership, and political participation are gaining relevance. Although these areas have fewer studies, their progressive presence in the academic agenda suggests an expansion of the field toward new domains requiring more complex theoretical frameworks and intersectional approaches.

The thematic analysis also identifies significant gaps in critical areas such as environmental justice with a gender focus, migration, and sexual diversity, which have limited representation in the reviewed literature. Identifying these gaps presents an opportunity for future research to broaden the analytical scope and foster a more inclusive approach to gender equity.

**Figure 4:**
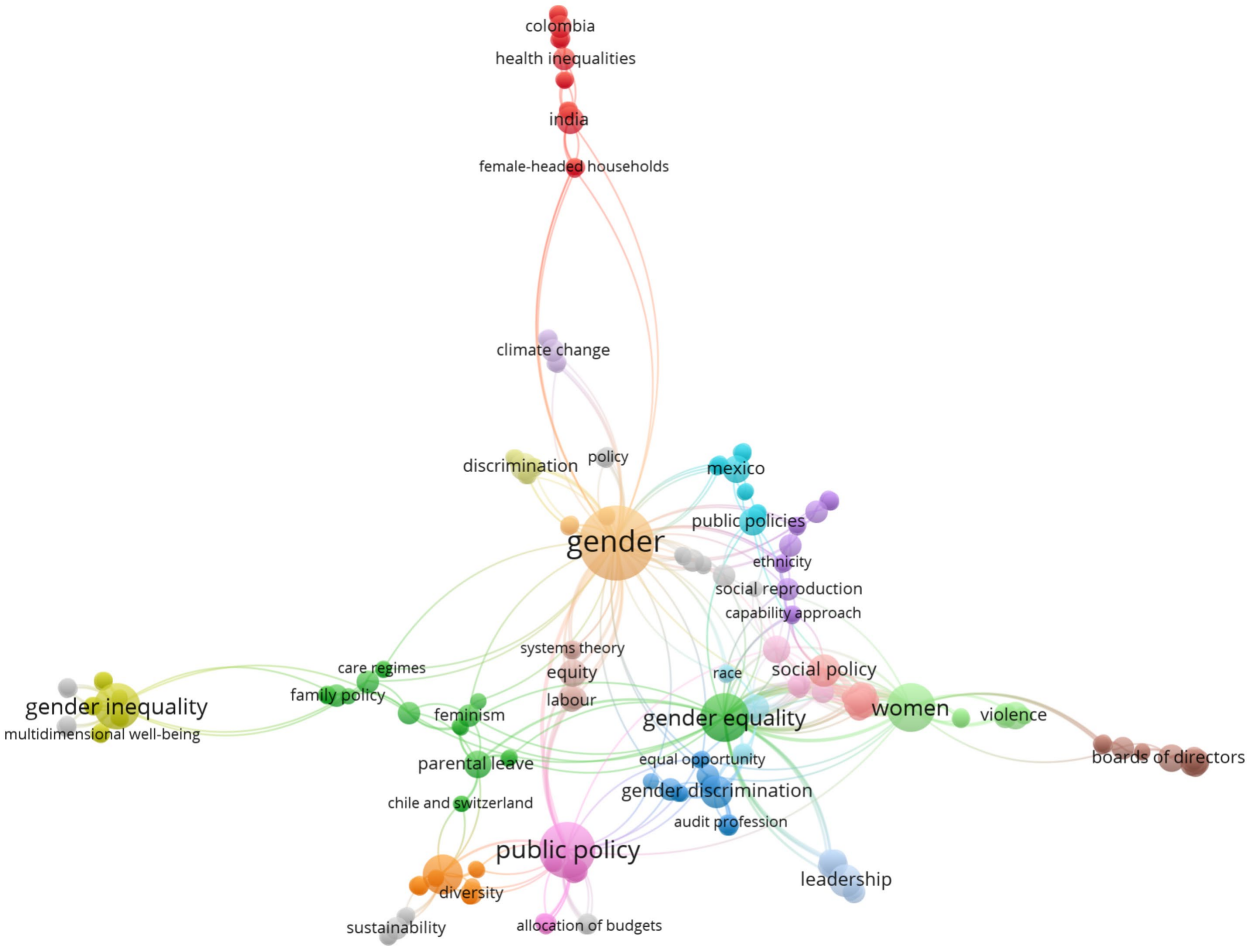
Distribution of thematic areas in gender equity research.

Collectively, Figure 4 illustrates that while consolidated hubs of scientific production exist in traditional gender equity topics, thematic diversification remains nascent. This finding underscores the need to promote editorial policies prioritizing inter- and transdisciplinary approaches to strengthen the field’s development.

The co-occurrence analysis of keywords reveals the most significant conceptual axes in recent gender equity research. The most recurrent terms include “gender,” “gender equity,” “discrimination,” “public policy,” “health,” “work,” “violence,” and “education,” confirming the cross-cutting nature of this issue.

The term “gender” is central in studies like those of Franc et al. (2018), Seguino (2011), Benería (2008), Lambert et al. (2021), and Matthews (2022), addressing inequality dynamics in health, religion, work, and social mobilization contexts. “Gender equity” is the main focus of research like Daly (2002), Forgues-Puccio and Lauw (2021), Plomien (2019), Hoyt and Kurtulus (2025), and Cowling et al. (2014), analyzing the impact of public policies and labor reforms across various geographical contexts.

“Gender discrimination” serves as a critical analytical framework in the works of Chakraborty (2020), Frías (2017), Naradech (2023), Welsh et al. (2017), and Phipps and Prieto (2021), which document structural barriers within the labor market and the manifestations of symbolic violence across various social contexts. In a complementary manner, the concept of “violence” is emphasized in studies by Contreras et al. (2023), Ayentimi et al. (2020), and Russi-Ardila (2020), underscoring the persistence of gender-based violence as a significant impediment to social and political participation.

In the domain of health, research conducted by Ariza-Ruiz et al. (2017), Franc et al. (2018), and Cowling et al. (2014) demonstrates the impact of gender inequalities on access to medical services, the quality of care, and public health indicators. This pattern is further corroborated by the studies of Saharso (2003) and Lambert et al. (2021), which examine the social determinants of health and their differential impact based on gender.

Research conducted by Linthon-Delgado and Méndez-Heras (2022), McGrath et al. (2022), Méndez-Suárez et al. (2025), Levasseur and Paterson (2016), and Chakraborty (2020) highlights significant barriers to equitable access to quality employment, persistent wage disparities, and structural challenges encountered by women in the labor market.

The concept of “education” is prominently featured in analyses such as those by Yates (1997), Arzú (2021), Mun et al. (2024), and Zetter et al. (2017), which investigate gender biases within curricula, school retention rates, and educational segregation resulting from gender stereotypes. These studies underscore the persistent challenge of achieving equity in educational access, particularly in rural and vulnerable settings.

In conclusion, the domain of “public policy” includes analyses such as those by Dekel and Elefant (2023), Javakhishvili and Jibladze (2018), Blofield and Martínez (2014), Güezmes García et al. (2023), and Mun et al. (2024), which examine the formulation, implementation, and constraints of governmental initiatives aimed at mitigating gender disparities.

The variety of keyword combinations underscores the intrinsic complexity of gender inequity and the progression towards more intersectional and transdisciplinary analytical frameworks. Research by Daly (2002), Lambert et al. (2021), Matthews (2022), Méndez-Suárez et al. (2025), Forgues-Puccio and Lauw (2021), McGrath et al. (2022), and Phipps and Prieto (2021) highlights the necessity of integrating social, economic, cultural, political, and health dimensions to effectively address gender inequalities in contemporary settings.

These findings underscore the importance of enhancing collaborative research strategies, promoting comparative analyses across regions, and incorporating equity perspectives in the design of public policies, educational systems, health services, labor markets, and political participation processes.

## Discussion

The findings of this systematic and bibliometric review indicate a notable increase in scientific output regarding gender equity since 2000. Nevertheless, this quantitative expansion is juxtaposed with a concentration of impacts within a limited number of pivotal studies, suggesting an uneven and dependent knowledge structure reliant on established references (Franc et al., 2018; Daly, 2002). This pattern is consistent with the observations of Akter et al. (2022), who noted that a substantial portion of gender-related publications tend to replicate theoretical frameworks without critically examining their contextual limitations or practical applicability.

These findings confirm the relevance of adopting an interdisciplinary search strategy, as gender equity research does not emerge from a single disciplinary domain but rather from interconnected policy arenas.

This review further underscores the disjunction between normative gender equity frameworks and their effective implementation. In contexts such as Georgia, anti-gender violence policies are implemented symbolically and hindered by insufficient resources and institutional commitment (Javakhishvili and Jibladze, 2018). This phenomenon corresponds with Merry’s (2005) concept of “performative compliance,” which highlights that the formal adoption of policies does not ensure transformative capacity. Similarly, Ramírez Rodríguez et al. (2015) emphasized the necessity of actively involving men in equality agendas, cautioning that unilateral approaches perpetuate imbalances rather than resolve them.

The findings within the labor domain indicate the persistence of structural impediments, such as the “glass ceiling” in Brazil (Proni and Proni, 2018) and occupational segregation in India (Chakraborty, 2020). These barriers not only limit women’s access to leadership roles, but also reinforce an economic system that disadvantages female labor, as posited by Benería (2008) from a feminist economics standpoint.

A critical issue is the insufficient application of intersectional methodologies. Although some studies incorporate variables such as ethnicity or gender identity (Arzú, 2021; Naradech, 2023), most adopt a binary and universalist perspective. This oversight is particularly concerning in the Global South context, where exclusionary dynamics are multifaceted and interconnected (Crenshaw, 1991; Saharso, 2003). The limited focus on dimensions such as disability, sexual orientation, and migration highlights a research field that remains constrained in diversity and critical depth.

The keyword analysis corroborates that the predominant conceptual axes—namely, GBV, education, “gender-based violence,” “education,” and “work” work—continue to shape the research agenda. Nevertheless, emerging themes such as climate change, artificial intelligence, and digital justice are underrepresented, highlighting areas that require an urgent gender perspective (Phipps and Prieto, 2021; Matthews, 2022). This observation aligns with Essig and Soparnot’s (2019) recommendation to renew theoretical frameworks to address contemporary social and technological transformation.

The international collaboration network reveals a significantly unequal distribution of knowledge. Countries in the Global North predominantly hold primary authorship roles, whereas those in the Global South are often involved as co-authors or secondary collaborators (Forgues-Puccio and Lauw, 2021). This pattern, previously criticized by Connell (2007), underscores epistemic inequalities that affect the representation and legitimacy of situated knowledge. Although initiatives exist to enhance intercontinental cooperation, the logic of extractivism persists in gender-related scientific productions.

From a network analysis perspective, the observed collaboration patterns suggest a structure characterized by central nodes with high connectivity, likely corresponding to institutions in the Global North. Although formal centrality and density metrics were not calculated, the visual clustering indicates a moderate level of modularity, with regional groupings and limited cross-regional integration. These patterns are consistent with previous bibliometric studies highlighting asymmetrical knowledge production structures.

In conclusion, this study reaffirms that advancing effective gender equity extends beyond merely increasing research volume. This necessitates transforming the structures of knowledge production, diversifying methodological approaches, and ensuring that policies yield tangible territorial impacts. Only through an intersectional, interdisciplinary, and geographically equitable approach can normative advancements be translated into sustainable structural change.

### Contributions to academic debate

This study makes a significant contribution to academic discourse on gender equity through multiple avenues. Initially, it provided a critical systematization of recent scientific literature, employing both bibliometric analysis and a systematic review in accordance with the PRISMA 2020 protocol, thereby enhancing methodological transparency and the reproducibility of results. In contrast to prior research that predominantly focuses on specific sectors, such as education or health, this study adopts an intersectional approach by integrating political, labor, health, and cultural dimensions. Furthermore, it underscores the persistent inequalities in the production of scientific knowledge, particularly highlighting the dominance of the Global North and marginalization of voices from the Global South. This perspective holds particular relevance for contemporary social studies, as it proposes actionable strategies not only for identifying existing gaps, but also for transforming the institutional structures that perpetuate these disparities.

### Methodological challenges in bibliometric analysis

Bibliometric analysis serves as a robust instrument to delineate research trends. However, several methodological challenges warrant recognition. A significant bias is the dominance of English in international databases, which constrains the representation of studies conducted in other languages and in regional contexts. This bias may skew perceptions of scientific contributions from regions such as Latin America, Africa, and Asia. Furthermore, although citation metrics are valuable for assessing visibility, they do not invariably reflect the quality, depth, or relevance of research, particularly in emerging or interdisciplinary fields. These limitations underscore the necessity to integrate quantitative methods with qualitative and contextual approaches to achieve more inclusive and representative analyses.

### Limitations

This study has several limitations. First, the selection of sources was confined to international databases, which, while ensuring rigor and quality, may have excluded pertinent local or regional research that addressed gender equity from contextualized perspectives.

Second, the use of specific search equations may have introduced a bias towards studies employing standardized terminology, potentially omitting works with emerging or interdisciplinary approaches that do not utilize the same keywords. Furthermore, the analysis primarily concentrated on bibliometric metrics such as publication volume or citations received. Although these metrics are useful for identifying general trends, they do not necessarily reflect the theoretical depth, methodological quality, or applicability of the analyzed studies.

Thirdly, a significant limitation is the exclusive use of the Scopus and Web of Science databases. It is well known that these platforms disproportionately index journals from the Global North, which may result in the under-representation of research produced in the Global South. Consequently, the geographical imbalances observed in knowledge production may partly reflect the coverage of the databases rather than actual research activity.

Another limitation pertains to the interpretation of international collaboration networks, as the factors explaining the observed regional asymmetries were not explored in depth. Finally, the study does not include an evaluation of the social impact of the reviewed policies, thereby limiting its capacity to establish direct connections between scientific knowledge and structural changes in gender equity.

### Practical implications

The study’s results offer several practical implications for policymakers, educational institutions, social organizations, and research teams.

First, gender equity policies must adopt a comprehensive approach, moving beyond legal and normative realms to address the structural and cultural dimensions perpetuating inequality.

Second, educational and labor institutions should implement active strategies to reduce gender gaps, such as promoting female leadership, redistributing care responsibilities, and strengthening wage equity.

At the public policy level, more participatory and adaptable designs are needed, enabling the evaluation of interventions’ real impact from a people-centered perspective, beyond mere compliance with quantitative indicators.

Finally, in the academic and scientific field, fostering situated, inclusive, and diverse knowledge production is crucial, amplifying Global South voices and promoting a more equitable circulation of knowledge, especially on topics related to gender, development, and social justice.

## Conclusion

This study represents a significant contribution to the structured analysis of scientific knowledge on gender equity, articulating an integrated vision of public policy, labor dynamics, and social movements from a systematic and bibliometric approach. Beyond listing findings, the methodological process allowed observation of how gender equity has evolved as a research field and which thematic and geographical configurations predominate in its study.

The review demonstrates that academic discourse on gender equity has consolidated as a cross-cutting concern across multiple disciplines. However, it also reveals that this field is marked by conceptual tensions, diverse methodological approaches, and asymmetries in the visibility of certain social realities. In this sense, scientific production not only reflects the state of gender equity but also shapes how it is understood, measured, and addressed.

One of the study’s most relevant contributions is the systematization of a knowledge base capable of guiding future institutional, educational, and governmental decisions. By integrating thematic trends, collaboration networks, and impact analyses, the study offers a useful cartography for formulating evidence-based policies and strengthening academic communities committed to social transformation.

Finally, the need to promote research processes that not only generate data or diagnoses but also nourish spaces for critical reflection, citizen participation, and institutional co-responsibility is reaffirmed. In an increasingly complex global context marked by challenges like digitalization, migration, and climate change, the gender perspective must occupy a strategic place in knowledge generation, not as an added category but as a structural axis for analysis, intervention, and justice.

## Data Availability

The original contributions presented in the study are included in the article/supplementary material, further inquiries can be directed to the corresponding author.

## References

[ref1] Ariza-Ruiz L. K. Espinosa-Menéndez M. J. Rodríguez-Hernández J. M. (2017). Challenges of menstruation in girls and adolescents from rural communities of the Colombian Pacific. Revista de Salud Pública 19, 833–841. doi: 10.15446/rsap.V19n6.71741, 30183839

[ref001] Akter M. Rahman M. Radicic D. (2022). Gender-aware framework in international entrepreneurship: how far developed? A Systematic Literature Review. Sustain. 14. doi: 10.3390/su142215326

[ref2] Arzú M. E. C. (2021). Diagnosis of racism and discrimination in Guatemala: qualitative and participatory methodology for the development of a public policy. Vibrant 18:e807. doi: 10.1590/1809-43412021v18a807

[ref3] Ayentimi D. T. Abadi H. A. Adjei B. Burgess J. (2020). Gender equity and inclusion in Ghana: good intentions, uneven progress. Labour Ind. 30, 66–84. doi: 10.1080/10301763.2019.1697486

[ref4] Benería L. (2008). The crisis of care, international migration, and public policy. Fem. Econ. 14, 1–21. doi: 10.1080/13545700802081984

[ref5] Blofield M. Martínez F. J. (2014). Work, family and public policy changes in Latin America: equity, maternalism and co-responsibility. CEPAL Rev. 114, 101–118. doi: 10.18356/1f42dd52-en

[ref6] Cañibano C. Fox M. F. Otamendi F. J. (2016). Gender and patterns of temporary mobility among researchers. Sci. Public Policy 43, 320–331. doi: 10.1093/scipol/scv042

[ref7] Chakraborty S. (2020). Gender wage differential in public and private sectors in India. Indian J. Labour Econ. 63, 765–780. doi: 10.1007/s41027-020-00246-1

[ref8] Cho J. Lee J. Kwon T. (2013). Gender exclusion in social security protection: evidence from Korea. Asian-Pac. Econ. Lit. 27, 62–78. doi: 10.1111/apel.12003

[ref9] Contreras H. Candia C. Troncoso R. Ferres L. Bravo L. Lepri B. . (2023). Linking physical violence to women’s mobility in Chile. EPJ Data Sci. 12:14. doi: 10.1140/epjds/s13688-023-00430-537215283

[ref002] Connell R. (2007). Southern Theory: Social Science And The Global Dynamics Of Knowledge. Polity Press.

[ref10] Cowling K. Dandona R. Dandona L. (2014). Social determinants of health in India: Progress and inequities across states. Int. J. Equity Health 13:Article 88. doi: 10.1186/s12939-014-0088-0, 25294304 PMC4201685

[ref003] Crenshaw K. (1991). Mapping the margins: Intersectionality, identity politics, and violence against women of color. Stanford Law Rev. 43, 1241–1299. doi: 10.2307/1229039

[ref11] Daly M. (2002). Care as a good for social policy. J. Soc. Policy 31, 251–270. doi: 10.1017/S0047279401006572

[ref12] Dekel T. Elefant L. (2023). The role of public policy in gender inequality in the arts in Israel. Isr. Stud. Rev. 38, 125–146. doi: 10.3167/isr.2023.380308

[ref13] Den Dulk L. Van Doorne-Huiskes A. Schippers J. (1996). Work-family arrangements and gender inequality in Europe. Women Manag. Rev. 11, 25–35. doi: 10.1108/09649429610122627

[ref004] Donthu N. Kumar S. Mukherjee D. Pandey N. Lim W. M. (2021). How to conduct a bibliometric analysis: an overview and guidelines. J. Bus. Res. 133, 285–296. doi: 10.1016/j.jbusres.2021.04.070

[ref005] Essig E. Soparnot R. (2019). Re-thinking gender inequality in the workplace—a framework from the male perspective. Management (France) 22, 373–410.

[ref14] Forgues-Puccio G. F. Lauw E. (2021). Gender inequality, corruption, and economic development. Rev. Dev. Econ. 25, 2133–2156. doi: 10.1111/rode.12793

[ref15] Franc K. A. Krecek R. C. Häsler B. N. Arenas-Gamboa A. M. (2018). Brucellosis remains a neglected disease in the developing world: a call for interdisciplinary action. BMC Public Health 18:125. doi: 10.1186/s12889-017-5016-y, 29325516 PMC5765637

[ref16] Frías S. M. (2017). Challenging the representation of intimate partner violence in Mexico: unidirectional, mutual violence and the role of male control. Partn. Abus. 8, 146–167. doi: 10.1891/1946-6560.8.2.146

[ref17] Gálvez-Muñoz L. Rodríguez-Modroño P. Domínguez-Serrano M. (2011). Work and time use by gender: a new clustering of European welfare systems. Feminist Econ. 17, 125–157. doi: 10.1080/13545701.2011.620975

[ref18] Güezmes García A. Bidegain Ponte N. Scuro M. L. (2023). Gender equality and the care society. CEPAL Rev. 141, 179–192.

[ref19] Hoyt E. Kurtulus F. A. (2025). Examining the effect of wrongful discharge laws on women’s occupational employment. Labour. 39, 101–128. doi: 10.1111/labr.12287

[ref20] Javakhishvili N. Jibladze G. (2018). Analysis of anti-domestic violence policy implementation in Georgia using contextual interaction theory (CIT). J. Soc. Policy 47, 317–334. doi: 10.1017/S0047279417000551

[ref21] Lambert A. Girard V. Guéraut E. (2021). Socio-economic impacts of COVID-19 on working mothers in France. Front. Sociol. 6:732580. doi: 10.3389/fsoc.2021.732580, 34977229 PMC8719263

[ref22] Lăzăroiu G. Rowland Z. Bartosova V. (2018). Gendered power disparities, misogynist violence, and women’s oppression: the #MeToo movement against workplace sexual harassment. Contemp. Read. Law Soc. Justice 10, 57–63. doi: 10.22381/CRLSJ10220184

[ref23] Levasseur K. Paterson S. (2016). Jack (and Jill?) of all trades – a Canadian case study of equity in apprenticeship supports. Soc. Policy Adm. 50, 520–539. doi: 10.1111/spol.12125

[ref24] Linthon-Delgado D. E. Méndez-Heras L. B. (2022). Decomposition of the gender wage gap in Ecuador. Rev. Mex. Econ. Finanzas Nueva Epoca 17, 1–25, e706. doi: 10.21919/remef.v17i1.706

[ref006] Lombardo E. Meier P. (2008). Framing gender equality in the European Union political discourse. Social Politics: International Studies in Gender, State & Society 15, 101–129. doi: 10.1093/sp/jxn001

[ref25] Matthews T. (2022). The politics of protest and gender: women riding the wings of resistance. Soc. Sci. 11:52. doi: 10.3390/socsci11020052

[ref007] Merry S. E. (2005). Human rights and gender violence: translating international law into local justice. University of Chicago Press. Available online at: https://press.uchicago.edu/ucp/books/book/chicago/H/bo3636543.html

[ref26] McGrath P. Acciaioli G. Millard A. Metzner E. Nešković V. V. Mathur C. (2022). The WCAA global survey of anthropological practice (2014–2018): reported findings. Vibrant 19:e701. doi: 10.1590/1809-43412022v19d701

[ref27] Méndez-Suárez M. Arilla R. Delbello L. (2025). The perception of effort as a driver of gender inequality: institutional and social insights for female entrepreneurship. Int. Entrep. Manag. J. 21:20. doi: 10.1007/s11365-024-01042-9

[ref28] Meng X. (1996). The economic position of women in Asia. Asian-Pac. Econ. Lit. 10, 23–41. doi: 10.1111/j.1467-8411.1996.tb00004.x

[ref29] Mun E. Vican S. Kelly E. L. (2024). Points of departure: family leave policy and women’s representation in management in U.S. workplaces. Soc. Forces 103, 520–549. doi: 10.1093/sf/soae080

[ref30] Naradech K. (2023). Factors of gender discrimination against transgender women in private organizations in Bangkok, Thailand. Humanit. Arts Soc. Sci. Stud. 23, 593–608.

[ref31] Page M. J. McKenzie J. E. Bossuyt P. M. Boutron I. Hoffmann T. C. Mulrow C. D. . (2021). The PRISMA 2020 statement: an updated guideline for reporting systematic reviews. BMJ 372:n71. doi: 10.1136/bmj.n71, 33782057 PMC8005924

[ref32] Phipps S. T. A. Prieto L. C. (2021). Leaning in: a historical perspective on influencing women’s leadership. J. Bus. Ethics 173, 245–259. doi: 10.1007/s10551-020-04566-6

[ref33] Plomien A. (2019). Gender inequality by design: does successful implementation of childcare policy deliver gender-just outcomes? Polic. Soc. 38, 643–662. doi: 10.1080/14494035.2019.1617513

[ref008] Proni T. T. D. R. W. Proni M. W. (2018). Gender discrimination in large companies in Brazil. Rev. Estud. Fem. 26. doi: 10.1590/1806-9584.2018v26n141780

[ref009] Ramírez Rodríguez J. C. de la Torre N. C. G. Hernández L. G. C. (2015). Building a public policy agenda gender of men in Mexico: prolegomenon. Masculinities and Social Change 4, 186–210. doi: 10.17583/MCS.2015.1514

[ref34] Russi-Ardila J. (2020). Legitimate guarantee of the public gender equity policy in an institution provider of health of Facatativa. Revista de Salud Pública 22, 521–526. doi: 10.15446/rsap.v22n5.81127, 36753220

[ref35] Saharso S. (2003). Culture, tolerance and gender: a contribution from the Netherlands. Eur. J. Womens Stud. 10, 7–27. doi: 10.1177/1350506803010001786

[ref36] Seguino S. (2011). Help or hindrance? Religion’s impact on gender inequality in attitudes and outcomes. World Dev. 39, 1308–1321. doi: 10.1016/j.worlddev.2010.12.004

[ref37] Shilton J. McGregor J. Tremaine M. (1996). Feminizing the boardroom: a study of the effects of corporatization on the number and status of women directors in New Zealand companies. Womens Manag. Rev. 11, 20–26. doi: 10.1108/09649429610117425

[ref38] Singh P. Singh V. K. Kanaujia A. (2025). Exploring the publication metadata fields in web of science, Scopus and dimensions: possibilities and ease of doing scientometric analysis. J. Scientometric Res. 13, 715–731. doi: 10.5530/jscires.20041144

[ref39] Vasudevan B. Chatterjee M. Sharma V. Sahdev R. (2025). Indexing of journals and indices of publications. Indian J. Radiol. Imaging 35, S148–S154. doi: 10.1055/s-0044-1800878, 39802723 PMC11717445

[ref9010] Walby S. (2020). Varieties of Gender Regimes. doi: 10.1093/sp/jxaa018

[ref40] Welsh D. H. B. Kaciak E. Minialai C. (2017). The influence of perceived management skills and perceived gender discrimination in launch decisions by women entrepreneurs. Int. Entrep. Manag. J. 13, 1–33. doi: 10.1007/s11365-015-0379-y

[ref41] Yates L. (1997). Gender equity and the boys debate: what sort of challenge is it? Br. J. Sociol. Educ. 18, 337–347. doi: 10.1080/0142569970180302

[ref42] Zetter B. C. Brambila C. G. Angón M. A. P. (2017). Gender desegregated analysis of Mexican inventors in patent applications under the patent cooperation treaty (PCT). Interciencia 42, 204–211.

